# Examining miR-196a, miR-196b and NF-κBIα gene expression in colitis mice model

**DOI:** 10.1186/s13568-025-01869-7

**Published:** 2025-07-03

**Authors:** Zahra Rafiezadeh, Shima Aboutalebian, Azar Baradaran, Laleh Hoveida, Seyed Hossein Hejazi

**Affiliations:** 1 Department of Microbiology, Fal.C., Islamic Azad University, Isfahan, Iran; 2 Department of Medical Parasitology and Mycology, School of Medicine, Isfahan University of Medical Sciences, Isfahan, Iran; 3 Mycology Reference Laboratory, Research Core Facilities, Isfahan University of Medical Sciences, Isfahan, Iran; 4 Department of Pathology, Isfahan University of Medical Sciences, Isfahan, Iran; 5 Infectious Diseases and Tropical Medicine Research Center, Isfahan University of Medical Sciences, Isfahan, Iran; 6 Department of Parasitology and Mycology, Skin Diseases and Leishmaniasis Research Center, School of Medicine, Isfahan University of Medical Sciences, Isfahan, Iran

**Keywords:** *Bifidobacterium bifidum*, Inflammatory bowel disease, Gut microbiota, miR-196a, miR-196b, Colitis

## Abstract

*Bifidobacterium bifidum* (*B. bifidum*) anti-inflammatory characteristics and ability to modify the gut microbiota make it a promising treatment option for Inflammatory Bowel Disease (IBD). The present study investigated the modulatory effects of *B. bifidum* on the expression of miR-196a, miR-196b, and *NF-κBIα* genes in a DSS-induced IBD mice model. Thirty male C57BL/6 mice were randomly assigned to five experimental groups (G1–G5). The induction of colitis used 3% DSS in drinking water for 8 days. The Disease Activity Index (DAI) was monitored, and mice were euthanized for further analysis on the 22nd day. The mice were gavaged with 2 × 10^9^ CFU/ml of *B. bifidum* per day. Histological grading of the colon tissues was done, and the levels of cytokines TNF-α and IL-6 were measured using ELISA. At the gene level, the expression of miR-196a, miR-196b, and NF-κBIα was analyzed by RT-PCR. Weight gain from days 11 to 17 in G3 and G4 with *B. bifidum* treatment pointed out its therapeutic benefits. Colon length, weight, and spleen weight were significantly decreased in G2 compared to G1 (*P* ≥ 0.0001). Histopathological examination revealed severe mucosal damage in G2, reduced inflammation in G3, fewer deep wounds in G4, and significant healing in G5. *B. bifidum*, especially in G5, significantly reduced IL-6 and TNF-α levels (*P* ≥ 0.0002). Gene expression analysis showed decreases in miR-196a and NF-κBIα and an increase in miR-196b, with G5 showing the most changes (*P* ≥ 0.0001). In conclusion, *B. bifidum* demonstrated significant anti-inflammatory effects in the DSS-induced IBD model by modulating miRNA expression and reducing key inflammatory cytokines.

## Introduction

The human gut microbiota comprises diverse microorganisms that are crucial to host physiological processes. These functions encompass manipulating and regulating the immune system, controlling intestinal maturation, adjusting mucosal physiology, and synthesizing essential substances like short-chain fatty acids and vitamins (Afzaal et al. 2022; Hou et al. 2022). Changing this community can profoundly affect human health, encompassing positive and negative outcomes. It has been suggested that the disturbance of the gut microbiota, also known as dysbiosis (Calleja-Conde et al. 2021), can play a substantial role in various pathological intestinal conditions such as obesity and malnutrition (Socol et al. 2022), as well as systemic diseases like diabetes and chronic inflammatory diseases, including inflammatory bowel disease (IBD), which comprises ulcerative colitis (UC) and Crohn’s disease (CD) (Franzosa et al. 2019). The development of IBD, can be attributed to alterations in the interaction between gut microbes and the immune system within the intestinal tract (Franzosa et al. 2019; Halfvarson et al. 2017).

The main medications used to treat IBD include immunosuppressive drugs like 5-aminosalicylic acid, corticosteroids, methotrexate, and thiopurines. However, the effectiveness of these drugs for treatment is usually limited, and there is a risk of severe adverse effects, especially during long-term treatment (Dong et al. 2022). Consequently, there is a requirement for novel strategies to prevent and clinically treat IBD. Probiotics provide a safer and more appealing therapeutic option for treating IBD. Probiotics are essential for safeguarding the intestinal mucosal barrier, balancing microbiota, and enhancing immune function, particularly for treating IBD (Ni et al. 2023). The symptoms of IBD can be relieved by certain strains of probiotics, particularly those found in the *Bifidobacterium* and *Lactobacillus* genera (Wang et al. 2014). Bifidobacteria are critical symbiotic organisms in the human intestines that help regulate metabolic and immunological functions (Duranti et al. 2016). Bifidobacteria strains are commonly used as live bio-therapeutics for their immunity-modulating properties and anti-inflammatory effects. In contrast, the absence or decline of *Bifidobacterium* species has been associated with several autoimmune and auto-inflammatory diseases in humans (Cristofori et al. 2021; Dong et al. 2022). For example, the levels of pro-inflammatory cytokines TNF-α, IL-6, IL-1β, IL-18, IL-22, and IL-9 are reduced in the colon homogenates of mice treated with *Bifidobacterium adolescentis* ATCC15703 (Gavzy et al. 2023).

Moreover, microRNA (miRNA) is gaining attention as a potential key player in the complex relationship between the microbiome and human health. The miRNAs are small, non-coding RNAs that regulate the expression of target genes (Yan et al. 2022). miRNA selectively target bacterial genes in the host intestinal environment, changing bacterial composition. This finding was an important step in understanding the microbiome-miRNA interaction. On the other hand, host miRNA expression may be affected by metabolites derived from microbiota, which may significantly influence host health (Liu et al. 2016). Therefore, dysregulation of the symbiotic relationship between miRNAs and microbiota can lead to various diseases, including neurological disorders, IBD, and colon cancer (Casado-Bedmar and Viennois 2022). Recent evidence indicates that gut microbiota influences intestinal miRNA, such as miR-194-5p, miR-148, miR-21, and miR-196, whose levels correlate with gut microbiota composition and inflammatory potential (Viennois et al. 2019).

The expression of miR-196 in IBD exhibits a pronounced contradiction. Studies have shown an increase in the levels of miR-196a and miR-196b in CD. However, in vitro experiments demonstrated no change in the expression levels of miR-196a or miR-196b when exposed to pro-inflammatory cytokines (IFN-γ) or infected with adherent invasive *E. coli* (AIEC) in HEK293 cells associated with CD (Brest et al. 2011; Papaconstantinou et al. 2017). Considering the raised challenges and the absence of in vivo studies on the expression of miR-196a and miR-196b, particularly in IBD, this study examines the expression levels of miR-196a, miR-196b and *NF-κBIα* in DSS-induced IBD. Furthermore, the study evaluates the associated inflammatory factors, including TNF-α and IL-6.

## Methods

### Ethical, Bacterial strains and culture conditions

Animal experiments were carried out at the School of Pharmacy, Isfahan University of Medical Sciences, Isfahan, Iran. The experimental protocol received approval from the Ethical Committee of Laboratory Animals at Azad University of Isfahan, Iran (approval number: IR.IAU.FALA.REC.1402.004). Under anaerobic conditions, *B. bifidum* ATCC 29521 was cultured for 48 h at 37 °C using the Anoxomat System AN2CTS (MART Microbiology BV, Lichtenvoorde, Netherlands) in an MRS agar medium supplemented with 0.05% l-cysteine. The fresh culture was used to prepare the daily bacterial dosage for gavage administration. For this purpose, the bacterial suspension obtained was centrifuged twice at 3500 rpm for 5 min in a buffer of PBS. The pellet was resuspended in sterile PBS, and each mouse was given 200 µL of it by gavage. The daily oral dosage was determined to be 2 × 10^9^ CFU/mL (Huang et al. 2023) by bacterial colony counting and spectrophotometry at a wavelength of 600 nm.

### Animals and probiotic treatment

This study included 30 male C57BL/6 mice, aged 7–9 weeks and weighing between 18 and 21 g, obtained from the Royan Institute in Isfahan, Iran (Dong et al. 2022; Festing and Altman 2002). For one week, the mice were given a standard diet and distilled water to adaptation. The mice were randomly assigned to five experimental groups, each with six mice. After that, the mice were placed in cages that followed a 12-h light–dark cycle. For improved analysis, groups were categorized into G1–G5. G1 consisted of healthy mice without intervention (Healthy Control). G2 comprised mice with DSS-induced IBD without treatment (Disease Model). G3 consisted of mice that were supplemented with *B. bifidum* during the first two weeks. In the third week, IBD was induced simultaneously with the co-administration of *B. bifidum*. Mice in G4 received *B. bifidum* for the first two weeks, then developed IBD in the last week without further *B. bifidum* supplementation. In G5, IBD was triggered in the initial week, and then *B. bifidum* was administered for two weeks.

### Colitis induction and assessment

For acute IBD induction, DSS powder was added to drinking water at a concentration of 3% for 8 days, and mice could drink freely. The DSS solution was replaced every day (Din et al. 2020). During treatment with DSS and *B. bifidum*, the disease activity index (DAI) was evaluated daily, considering factors such as weight loss, stool consistency, and hematochezia. In the stool, blood was detected using an occult blood (OB) kit (Aria Mabna Tashkhis, Tehran, Iran). To prevent hemolysis and facilitate serum separation, blood samples obtained from the eye were refrigerated for one hour before being centrifuged at 12,000 rpm for 5 min. The extracted serum was stored at − 20 °C until inflammatory cytokines were determined. After the experiment was finished, the mice were euthanized using 150 mg/kg of pentobarbital. Following the dissection of the mice on the 22nd day, we assessed the DAI by measuring the weight and length of the colon and spleen.

### Histological assessment

Initially, colon tissue samples were fixed in 10% formaldehyde at 4 °C for 24 h and then dehydrated using ethanol series. Subsequently, ethanol was replaced by xylene.

Sections measuring 1–2 microns in thickness were cut from the blocks using a microtome. These sections were then stained with hematoxylin–eosin and examined under a light microscope equipped with a camera for routine histological analysis. The severity of colonic histological injury was evaluated based on several criteria, including inflammation, mucosal damage, crypt damage, pathological alterations, and goblet cell loss (Koelink et al. 2018).

### Cytokine analysis

The colonic samples were homogenized in ice-cold PBS solution (1:9, w/v). The resulting homogenate was centrifuged at 3500 rpm for 10 min, and the clear supernatant was collected for processing inflammatory cytokines. The concentrations of TNF-α and IL-6 in both the colon and serum of the mice were measured in duplicate using a commercially available kit (R & D Systems, Minneapolis, USA), following the manufacturer's instructions.

### Gene expression analysis by RT-PCR

The miRNA was extracted from each colonic tissue sample using the AnaCell mmu-mir-196a kit (AnaCell, Tehran, Iran) in accordance with the manufacturer's instructions. The extracted RNA samples were then quantified using the Thermo Scientific NanoDrop™ 2000 Spectrophotometer (Thermo Fisher Scientific, Waltham, MA, USA) to determine RNA concentration and purity. To synthesize complementary DNA (cDNA), 1 μg of total RNA from each sample was used. The cDNA synthesis was performed using the cDNA Synthesis Kit (AnaCell, Tehran, Iran), following the manufacturer's protocol. The reaction mix was prepared and incubated as per the recommended thermal cycling conditions.

Quantitative real-time PCR (RT-PCR) was conducted using SYBR® Premix (Metabion, Planegg, Germany) to evaluate the expression levels of miR-196a, miR-196b, and *NF-κBIα*. The analysis was performed on an ABI 7500 FAST system (Applied Biosystems, Foster City, CA, USA). Each 20 μL PCR reaction consisted of 10 μL SYBR® Premix, 0.8 μL of each forward and reverse primer (10 μM), 2 μL of cDNA template, and 6.4 μL of nuclease-free water. The PCR conditions included an initial denaturation step at 95 °C for 10 min, followed by 40 cycles of denaturation at 95 °C for 15 s and annealing/extension at 60 °C for 1 min. Beta-Actin was used as the internal control to normalize the expression levels of the target genes, and the relative expression levels were quantified using the 2^−ΔΔCt^ method. Table 1 contains a list of all the primer details used in this study.

**Table 1:** The primers used for RT-PCR

Gene	Sequences 5′- 3′	References
*miRNA-196a*	Forward: 5′-CGTCAGAAGGAATGATGCACAG-3′	Shahabi et al. (2020)
	Reverse: 5′-ACCTGCGTAGGTAGTTTCATGT-3′	
*miRNA-196b*	Forward: 5′-TAGGTACCACTTTATCCCGTTCACCA -3'	Xu and Gu (2017)
	Reverse: 5′-ATCTCGAGGCAGGGAGAGAGGAATAA-3′	
*NF-κBIα*	Forward: 5′-ACCTGGTGTCACTCCTGTTG-3′	Kazemian et al. (2022)
	Reverse: 5′-GCTCTCCTCATCCTCACTCTC-3′	
*beta-Actin*	Forward: 5′-AAGGCCAACCGCGAGAAGAT-3′	Chen et al. (2020)
	Reverse: 5′-GCCAGAGGCGTACAGGGATA-3′	

### Statistical analysis

The data are expressed as mean ± standard deviation (SD). Statistical significance in data comparisons was assessed by conducting a one-way analysis of variance (ANOVA) test using GraphPad Prism 9.0 statistical software. A *P* value of less than 0.05 was considered statistically significant.

## Results

### DAI assay

**Fig. 1:**
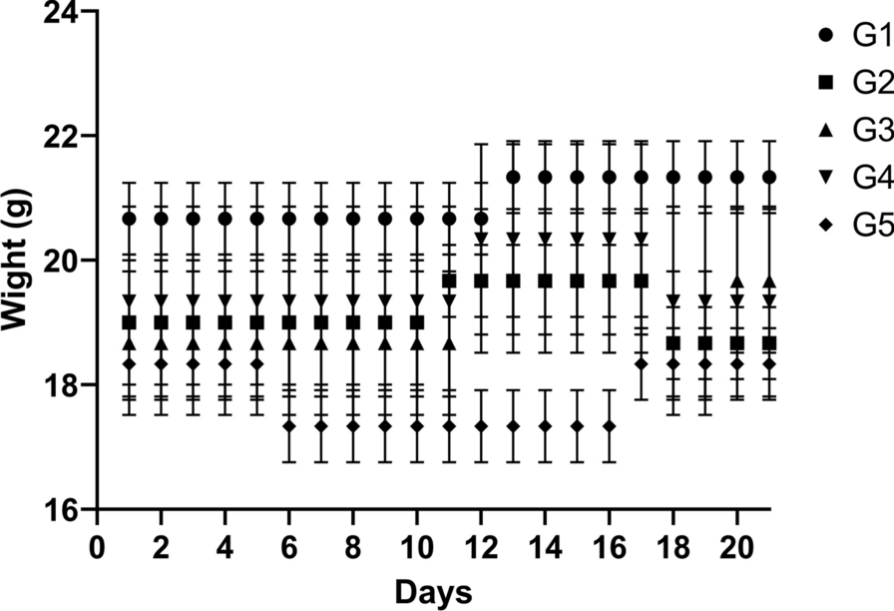
Comparison of weight changes in mice throughout a 21-day study in different groups

According to the results, the initiation of IBD in mice leads to weight loss, changes in fecal consistency, and the appearance of blood in their feces within the groups administered DSS. Compared to the G1 group, the G2 group significantly decreased in weight (*P* ≥ 0.0001). Between day 11 and day 17, mice in the G3 group exhibited weight gain, suggesting that *B. bifidum* conferred both therapeutic and preventive advantages. Similarly, the G4 group demonstrated a weight increase in mice from day 11 to day 17, implying a protective effect of *B. bifidum*. Weight loss in the G4 group from days 18 to 21 was slightly higher compared with the G3 group, but this can be attributed to the fact that the G4 group did not receive *B. bifidum* in the last week. In the G5 group, there was a decline in the weight of mice from the fifth to the sixteenth day, signifying the induction of colitis. However, from the sixteenth to the twenty-first day, there was an increase in the weight of mice (*P* ≥ 0.0001), indicating the positive therapeutic effects of *B. bifidum* (Fig. 1).

**Fig. 2:**
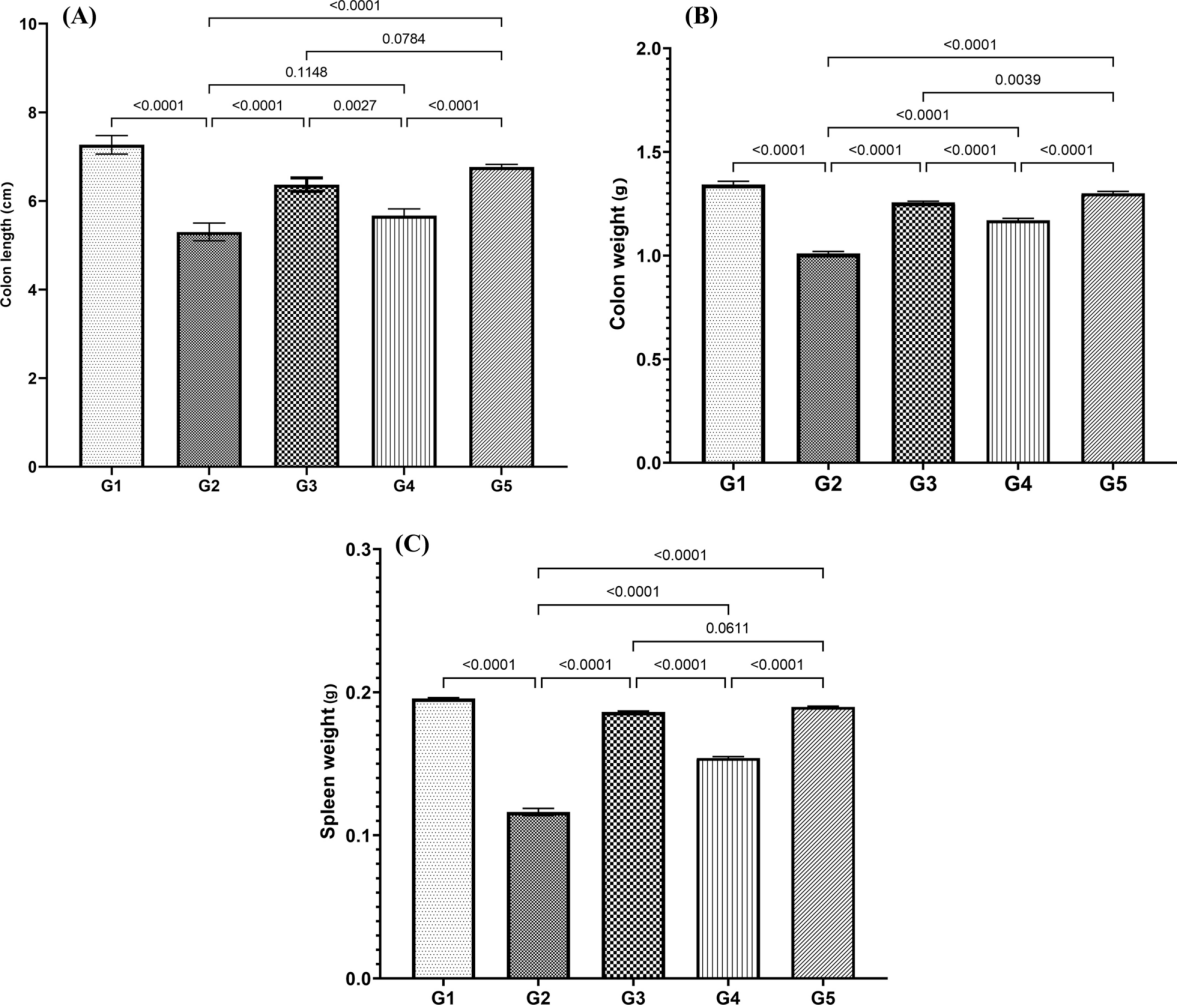
Comparison of the colon size (**A**), colon weight index (**B**), and spleen weight **C** after IBD induction among different study groups. Results were analyzed using ANOVA, with non-significant differences indicated as (*P* > 0.05)

Furthermore, the findings suggest that, following the induction of IBD, a significant decrease in colon length, colon weight, and spleen weight was observed in the G2 group compared to the G1 group (*P* ≥ 0.0001). Significant differences were observed in colon weight (Fig. 2A) and spleen weight (Fig. 2B) among the different groups. Compared to the G2 group, the G3 group demonstrated a significant increase (*P* ≥ 0.0001) in colon weight after receiving pre-treatment with *B. bifidum* for 14 days, followed by treatment during the last week along with DSS. Pre-treatment alone with *B. bifidum* led to a more significant (*P* ≥ 0.0001) decrease in colon weight in the G4 group than in the G3 group. A very slight weight loss was observed in the colon of the G5 group, and the colon's weight was similar to that of the G1 and G3 groups. The colon length exhibited considerable diversity among the groups, with the G2 group displaying the shortest colon, followed by the G3, G4, and G5 groups, respectively, while the G1 group functioned as the control (Fig. 2C, D)

### Histopathological examination

**Fig. 3:**
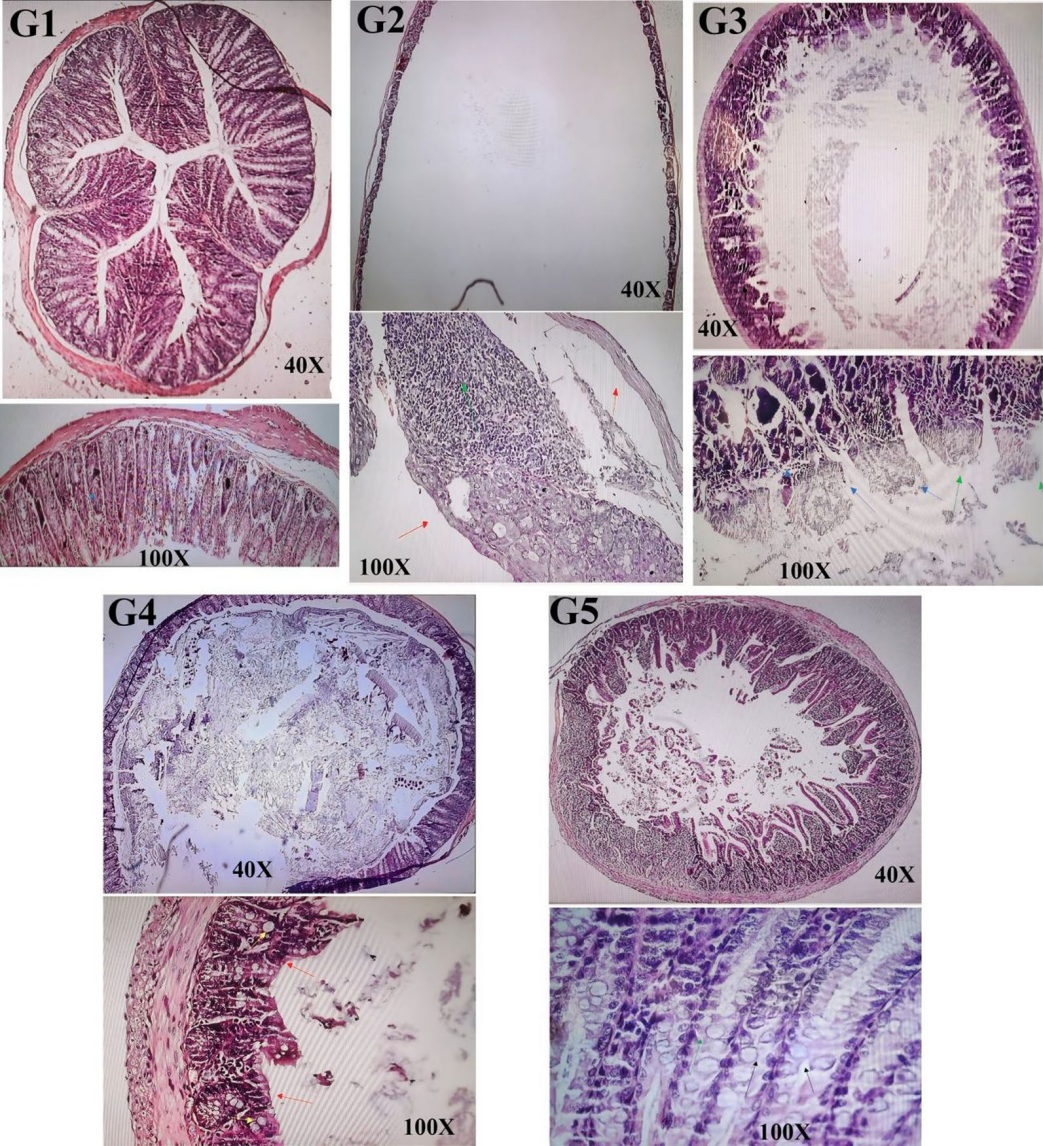
The *B. bifidum* reduces the severity of IBD. Representative colon images stained with H&E: G1: Normal histological features with well-defined mucosal folds. G2: Severe loss of mucosal epithelium across the intestinal wall with extensive inflammatory infiltration into the muscularis layer. G3: inflammation and superficial wounds were diminished, with no deep wounds observed and reduced swelling. G4:Mostly superficial or erosive wounds, with fewer deep ulcers compared to the G2 group. G5: Complete recovery of lining epithelial cells, normal villus height, no deep wounds, and a marked reduction in surface erosions, indicating active healing and repair

According to the pathology findings, in the G1 group the mucosa, submucosa, muscular layer, and serosa of the colon all appear to be in a state of structural health and devoid of any observed damage (Fig. 3). The G2 group exhibits a notable absence of mucosal epithelium across the entire intestinal wall, accompanied by pronounced inflammatory infiltration extending to the muscular layer. Ulceration is widespread throughout the intestinal wall. Thinning of the mucosal epithelium is evident throughout the colon, concomitant with an increased presence of neutrophils. Glandular structures display aberrant morphology indicative of destruction, with inflammation extending to the deep layers of the intestinal wall. Enlargement of glandular nuclei and observable cellular and nucleolar changes are apparent. Additionally, edema and inflammation of intestinal crypts, alongside dysplastic alterations, are observed. In G3 group, inflammation and superficial wounds were diminished, with no deep wounds observed and reduced swelling. Mucous folds exhibited fewer irregularities compared to G2 group, resulting in a significant reduction in overall irregularities. Goblet cell reduction was less evident compared to G2 group, suggesting lesser damage to the clone overall. In G4 group, there is noticeable mucosal epithelial damage accompanied by severe inflammation. While most wounds are superficial or erosive, there are fewer deep wounds compared to the G2 group, indicating the positive impact of pretreatment with *B. bifidum*. Although the smoothing of the folds is not as pronounced as in the second group, they still exhibit some irregularities, albeit less damaged than those in the G2 group. In the G5 group, the villi have resumed their typical height, and the epithelial cells lining the area are fully functional. Notably, no deep wounds were observed, and a notable decrease in surface wounds or erogenous wounds was noted, indicative of ongoing wound healing and repair processes. Additionally, a discernible increase in goblet cell count was observed relative to the comparison G2 group.

### Cytokines assay

**Fig. 4:**
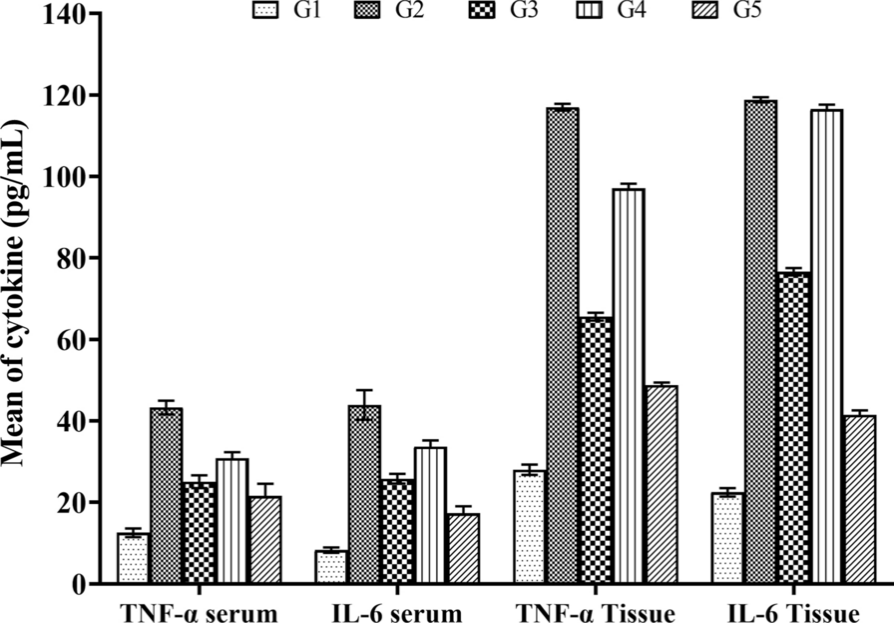
Comparison of the levels of cytokines IL-6 and TNF-α in the serum and intestinal tissue among the study groups

Statistical analysis reveals that administering *B. bifidum*, particularly in the G5 group, notably decreased IL-6 and TNF-α expression levels compared to the G2 group, resulting in a significant amelioration of IBD (*P* ≥ 0.0002). Additionally, examination of cytokine levels in intestinal tissue, as illustrated in Figs. 4, demonstrated the most notable therapeutic outcomes in the G3 and G5 cohorts (*P* ≥ 0.0001), whereas the G4 group did not exhibit a significant reduction in IL-6 cytokine levels (*P* ≥ 0.2669). The G5 group experienced the most notable decrease in IL-6 and TNF-α levels.

### Expression of *miR-196a*, *miR-196b*, and *NF-κBIα* genes

**Fig. 5:**
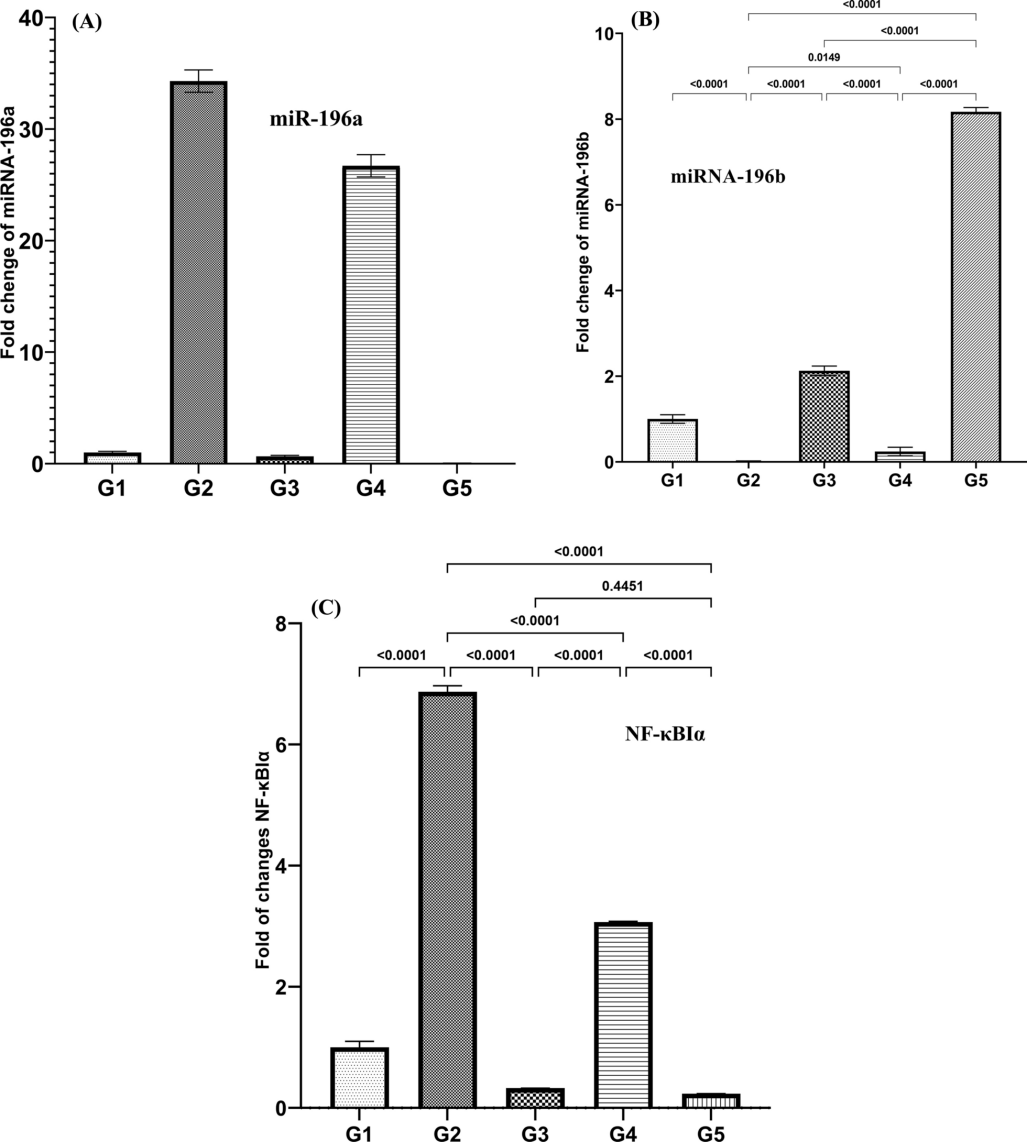
Expression of miR-196a, miR-196b, and NF-κBIα genes by RT-PCR. Results were analyzed using ANOVA, with non-significant differences indicated as (*P* > 0.05)

Statistical analysis reveals that treatment with *B. bifidum* in all groups resulted in a significant decrease in the expression levels of *miR-196a* compared to the G2 group *(P* ≥ 0.0001), thereby contributing to the improvement of IBD symptoms. The most significant decrease in *miR-196a* levels was noted in the G5 group (Fig. 5). In contrast to *miR-196a*, *miR-196b* exhibited a substantial increase in expression across all treatment groups compared to the G2 group, with particularly noteworthy elevation observed in the G5 group, reaching a 400-fold increase. Furthermore, analysis of the expression level of the cytokine *NF-κBIα* indicates that a significant reduction in its level (*P* ≥ 0.0001) was observed in all three treatment and pre-treatment groups, including the G3, G4, and G5.

## Discussion

miRNAs emerging as novel biomarkers and therapeutic targets offer promising avenues for treating IBD. The regulatory role of microRNAs in tight junction proteins and modulation of epithelial/endothelial barrier functions, as well as inflammation in IBD, has been well recognized. In addition, the differential expression of microRNAs that has been observed between healthy and IBD patients provides additional evidence for their importance in the pathogenesis of the disease (Jung et al. 2021). According to recent studies, their results suggested that miRNAs could have a negative or positive affect on the occurrence and development of IBD. Investigations into the miRNA expression profile within tissues of individuals with IBD have yielded somewhat conflicting and challenging-to-interpret results. Numerous studies examining miRNA involvement in IBD have reported both upregulation and downregulation, highlighting the complexity of miRNA dynamics in this context (Casado-Bedmar and Viennois 2022; Dalal and Kwon 2010).

To our knowledge, this study is the first in vivo examination of miR-196a and miR-196b expressions in IBD, but previous research has focused on other disorders, such as cancers. For example, miR-196a and miR-196b have been implicated in the modulation of inflammatory responses and epithelial barrier integrity, suggesting their involvement in key pathogenic mechanisms underlying IBD. Studies have shown an increase in the levels of miR-196a and miR-196b in CD. However, in vitro experiments demonstrated no change in the expression levels of miR-196a or miR-196b in other studies (Brest et al. 2011; Krishnachaitanya et al. 2022; Papaconstantinou et al. 2017). Considering the raised challenges and limited in vivo studies on the expression of miR-196a and miR-196b, particularly in IBD, this study examines the expression levels of miR-196a and miR-196b in DSS-induced IBD.

The present study demonstrates that treatment with *B. bifidum* led to a significant decrease in the expression levels of miR-196a across all groups compared to the G2 group. Conversely, miR-196b showed a substantial increase in expression across all treatment groups compared to the G2 group, with a particularly remarkable elevation observed in the G5 group, reaching a 400-fold increase (*P* ≥ 0.0001). A clinical study revealed notable upregulation of circulating miR-196a and miR-196b in patients with oral pre-cancer lesions, showing increases of 5.9-fold and 14.8-fold, respectively (*P* < 0.01). Similarly, elevated levels of miR-196a and miR-196b were observed in patients diagnosed with oral cancer, exhibiting increases of 9.3-fold and 17.0-fold, respectively (Lu et al. 2015). Contrary to expectations, this study found that miRNA-196a was upregulated while miRNA-196b was downregulated in the experimental group’s DSS-induced IBD (G2). In a study conducted by Saúl Álvarez et al., elevated levels of miR-196b were identified in 95% of primary tumors and precancerous lesions, although no significant variances were noted between non-progressing and progressing dysplasia. Moreover, increased levels of both miR-196a and miR-196b were reliably detected in saliva samples collected from patients with head and neck squamous cell carcinoma (Álvarez-Teijeiro et al. 2017). Despite differing by just a single nucleotide in maturity, both miR-196a and miR-196b exhibit diverse expression levels and functional implications across a wide range of diseases. Although research has shown conflicting results, some tumor types (including melanoma, astrocytoma, osteosarcoma, and myeloma) and experimental investigations have linked miR-196 downregulation to tumor-suppressing effects (Coira et al. 2022; Dioguardi et al. 2022; Guan et al. 2010; Pazzaglia et al. 2015).

The NF-κBIα activation has been implicated in the pathogenesis of IBD. In particular, NF-κBIα activation in intestinal epithelial cells and immune cells leads to the production of pro-inflammatory cytokines, chemokines, and adhesion molecules, which contribute to the recruitment and activation of immune cells in the gut mucosa (Papoutsopoulou et al. 2019). This chronic inflammation perpetuates tissue damage and disrupts the intestinal barrier function, further exacerbating the disease process in IBD (Aggeletopoulou et al. 2024; Merga et al. 2016). Therefore, inhibition NF-κBIα has been explored as a therapeutic strategy for IBD to dampen excessive inflammation and restore intestinal homeostasis. Various approaches targeting NF-κBIα signaling, including small molecule inhibitors, biologics, probiotics and dietary interventions, have shown promise in preclinical and clinical studies for the treatment of IBD. The analysis of NF-κBIα expression using real-time PCR revealed a notable decrease in all three treatment and pretreatment groups (G3, G4, and G5) (*P* ≥ 0.0001). Our investigation has demonstrated that *B. bifidum* effectively suppresses the expression of NF-κBIα.

IL-6 and TNF-α play crucial roles in the pathogenesis of IBD by promoting inflammation and immune dysregulation in the gastrointestinal tract. Targeting these cytokines has been a major therapeutic strategy for managing IBD, leading to the development of biologic therapies that specifically inhibit IL-6 or TNF-α signaling pathways (Alhendi and Naser 2023; Shahini and Shahini 2023). In the G2 group, our findings revealed a significant increase in IL-6 and TNF-α levels, indicating successful induction of IBD by DSS. In the treatment and pre-treatment groups receiving *B. bifidum*, particularly in the G3 and G5 groups, there was a significant decrease in the concentration of these cytokines. This reduction in the levels of the investigated cytokines may be attributed to the anti-inflammatory properties of *B. bifidum*. The use of anti-inflammatory represents a promising therapeutic approach for the treatment of IBD. By targeting key inflammatory pathways and cytokines such as IL-6 and TNF-α, these agents aim to suppress inflammation, alleviate symptoms, and improve the quality of life for individuals with IBD. While biologic therapies targeting IL-6 or TNF-α have shown efficacy in many patients, further research is needed to optimize treatment strategies, identify new therapeutic targets, and personalize therapy to individual patient needs. Additionally, ongoing efforts to develop novel anti-inflammatory agents and combination therapies hold great promise for advancing the management of IBD and achieving better outcomes for patients in the future. in summary, this study demonstrates that treatment with *B. bifidum* is effective in reducing inflammation associated with IBD. Also, our findings indicate that the downregulation of miR-196a or the upregulation of miR-196b effectively inhibited the progression of IBD. These results unveil novel targets for the prognosis and therapeutic intervention of IBD. Hence, comprehensive studies on miRNAs, encompassing investigations into their interactions with other genes, their impact on protein expression, and site-specific miRNA expression profiling, are imperative prior to embarking on clinical trials and considering potential therapeutic applications.

## Data Availability

The authors confirm that the data supporting the findings of this study are available within the article materials.
